# Influence of timing (pre-puberty or skeletal maturity) of ovariohysterectomy on mRNA levels in corneal tissues of female rabbits

**Published:** 2008-03-06

**Authors:** Yamini Achari, Carol R. Reno, Helen Tsao, Douglas W. Morck, David A. Hart

**Affiliations:** McCaig Institute for Bone and Joint Health, Faculty of Medicine, University of Calgary, Calgary, Alberta, Canada

## Abstract

**Purpose:**

Corneal thickness and curvature are reported to be influenced by hormonal changes associated with menstrual cycle, pregnancy, or menopause. However, the molecular mechanisms leading to these alterations are not clearly understood. The present study focuses on gene expression patterns (mRNA levels) in corneal tissues following surgically induced menopause in an animal model. The impact of lower hormone levels on mRNA levels in corneal tissues after pre-puberty ovariohysterectomy (OVX) was compared to that in skeletally mature adult animals.

**Methods:**

Skeletally mature adult female rabbits were either left unoperated (control) or were subjected to OVX at 54 weeks of age using an approved protocol. The central (~6 mm) and the peripheral corneal tissues were harvested from normal and OVX rabbits eight weeks after surgery. In a second study, young sexually immature rabbits at eight weeks of age were subjected to OVX and corneal tissues were collected when the animals were 22 and 32 weeks of age. In both experiments, RNA was isolated from corneal tissues and RT–PCR was used to assess mRNA levels for several relevant molecules.

**Results:**

When mature animals were examined eight weeks after OVX, mRNA levels for molecules such as the estrogen receptor, decorin, collagen I, collagen V, and several growth factors were found to be significantly decreased in central corneal tissues. Interestingly, no corresponding changes in mRNA levels were observed for these same molecules in peripheral corneal tissues. When young, pre-pubertal animals were subjected to OVX, mRNA levels were found to be mainly unchanged for the OVX animals at 22 weeks of age i.e., after 14 weeks of low hormone conditions. However, significant decreases in mRNA levels for a similar subset of molecules were observed when the animals were at least 32 weeks of age, i.e., after 24 weeks of a low hormone environment. Examination of peripheral corneal tissues did not show significant changes in mRNA levels due to OVX at either 22 or 32 weeks of age except for collagens I and V at 32 weeks of age.

**Conclusions:**

These results indicate significant alterations in mRNA levels in the central corneal tissues of rabbits following OVX. Interestingly, peripheral corneal tissues show very little alteration in mRNA levels for the same molecules. Furthermore, OVX had a more rapid impact on mRNA levels in mature animals than in skeletally immature animals. Thus, loss of hormone producing tissues during growth and maturation apparently delayed the impact of hormone removal compared to loss after maturity had been attained and growth stimuli are likely absent. Therefore, specific areas of the cornea are more responsive to hormone levels than others. The impact of the loss of hormones is influenced by the maturation state of the rabbit, but mRNA levels for a similar subset of genes are affected by OVX in both age groups.

## Introduction

It is well known that the normal process of aging leads to alterations in visual function. Several reports suggest that women exhibit a higher degree of changes to ocular tissues when compared to men. Ophthalmic changes such as increased intraocular pressure [1], decline in tear film function [2], alterations in trabecular meshwork [3], and reduction of visual performances such as contrast sensitivity [4] are all found to be higher in aging women. In addition, post-menopausal and/or elderly women exhibit a higher incidence of age-related macular degeneration (AMD), idiopathic full thickness macular hole, and cataract [5-7]. Such studies suggest that fluctuations in hormone levels in women might contribute to the onset and progression of ocular diseases.

Women experience fluctuations in hormonal levels throughout their life span in association with puberty, menstrual cycles, pregnancy, and menopause. The presence of sex steroid receptors such as those for estrogen, progesterone, and androgen have been demonstrated in the ocular tissues of mouse, humans, and rabbits [8-10] suggesting that these tissues may be hormone responsive. The cornea is a highly intricate collagenous structure which functions as the protective cover for the eye and contributes to a major part of the eye’s focusing power via refraction of light [11-13]. Variations in corneal thickness have been associated with hormonal changes during the menstrual cycle [14]. Furthermore, hormonal changes associated with pregnancy or menopause hve also been reported to influence corneal thickness and curvature [15-17].

Besides structural changes such as alterations in thickness or curvature of cornea, hormonal changes may also lead to the onset and development of a range of ocular ailments. Women are at a higher risk for developing age-related macular degeneration, which is the leading cause of blindness in Caucasian populations [18]. The onset of this disorder is closely associated with menopause, especially as premature onset of menopause has been shown to lead to its untimely development [19].

**Table 1:** Primers used in reverse transcription polymerase chain reaction experiments.

**Gene**	**Forward primer**	**Reverse primer**	**Tm (°C)**	**bp**	**Source**
ER	GTGTCTGTGATCTTGTCC	CTCCATGATCAGGTCCAC	60	341	GenBank X73067
PR	CCACAGTACAGCTTCGAGTC	CCTGCATAGATCACATCTGG	60	431	GenBank M14547
Col-I	GATGCGTTCCAGTTCGAGTA	GGTCTTCCGGTGGTCTTGTA	55	312	Kao, W.W. Personal communication
Col-III	TTATAAACCAACCTCTTCCT	TATTATAGCACCATTGAGAC	55	255	Pelliniemi, L., Vuorio, E., Personal communication
Col-V	GAGGAGAACCAGGAATAACC	GCACCTTTCTCTCCGATGCC	55	215	Bluteau, G., Personal communication
Biglycan	GATGGCCTGAAGCTCAA	GGTTGTTGAAGAGGCTG	60	406	GenBank AF020290
Decorin	TGTGGACAATGGTTCTCTGG	CCACATTGCAGTTAGGTTCC	55/60	419	GenBank S76584
FGF	TACAACTTCAAGCAGAAGAG	CAGCTCTTAGCAGACATTGG	55	282	GenBank X04432, X04433
CTGF	TTGTAGCTGATCAGTCTTTCCAC	CAACTAAAAAGGTGCAAACATGTAA	60	119	GenBank NM_001901
IGF-1	GCATCCTGTCCTCCTCGCAT	GTCTTGGGCATGTCGGTGTG	60	322	consensus sequences from GenBank sequences
NGF	GGTGCATAGCGTAATGTCCA	TTGCTCCTGTGAGTCCTGTT	55	372	GenBank XM_227525
TGF-β	CGGCAGCTGTACATTGACTT	AGCGCACGATCATGTTGGAC	60	271	GenBank AF000133
CathK	AGCTGGGGAGAAAGCTGGGGAAACAAAG	AGGCACAAACAAATGGGGAAACCAAACA	65	245	GenBank D14036
OPN	CCGATGACTCTCACCACTCC	CCTCTTCACTCTTCGGCTCG	60	549	GenBank D11411
TSP-1	AGTGACTCAGCAGATGATGG	CACAGGTGACAGAGCAGATG	65	403	GenBank D16544
VEGF	GGAGTACCCTGATGAGATCGA	CTTTGGTCTGCATTCACATTTGT	60	211	GenBank AF022179
β-Actin	TGCTTCTAGGCGGACTGTTA	CGTCACATGGCATCTCACGA	55	314	GenBank U07786

The indicated forward and reverse primers for the molecules indicated in Lane 1 were validated for use in the rabbit. The size of the expected PCR product for each molecule is given in base pairs (Lane 5). The original sources of information used to develop the indicated primer sets is given in Lane 6. Several of the indicated primer sets have been used in previous rabbit studies [20,27].

Although several reports suggest that the cornea is a highly responsive tissue to the fluctuating levels of sex hormones, a detailed molecular analysis of the effects of the hormones on gene expression in the corneal tissues is still somewhat lacking. Therefore, the present study was undertaken to focus on mRNA levels for relevant molecules in cornea such as hormone receptors (estrogen receptor, progesterone receptor), matrix molecules (collagen I, III, V, biglycan, and decorin), several pertinent growth factors (bFGF, CTGF, TGF-β, NGF, IGF-I, and VEGF), and molecules implicated in pathologic processes (cathepsin K, osteopontin, and thrombospodin-1) following surgical menopause (e.g., ovariohysterectomy) in an animal model. This study was based on the hypothesis that mRNA levels for certain genes will change as a result of the hormonal decline which is a characteristic feature of menopause, and additionally, the pre-pupillary or central cornea will exhibit a gene expression pattern which is different from the peri-pupillary or peripheral region of the cornea. Furthermore, the impact of removal of sex hormones before the attainment of puberty was compared to the loss of sex hormones in later stages of life as a skeletally mature adult. The ability to envisage the corneal response to changing hormonal levels may be of clinical significance in predicting vision changes with age, particularly in females.

**Figure 1:**
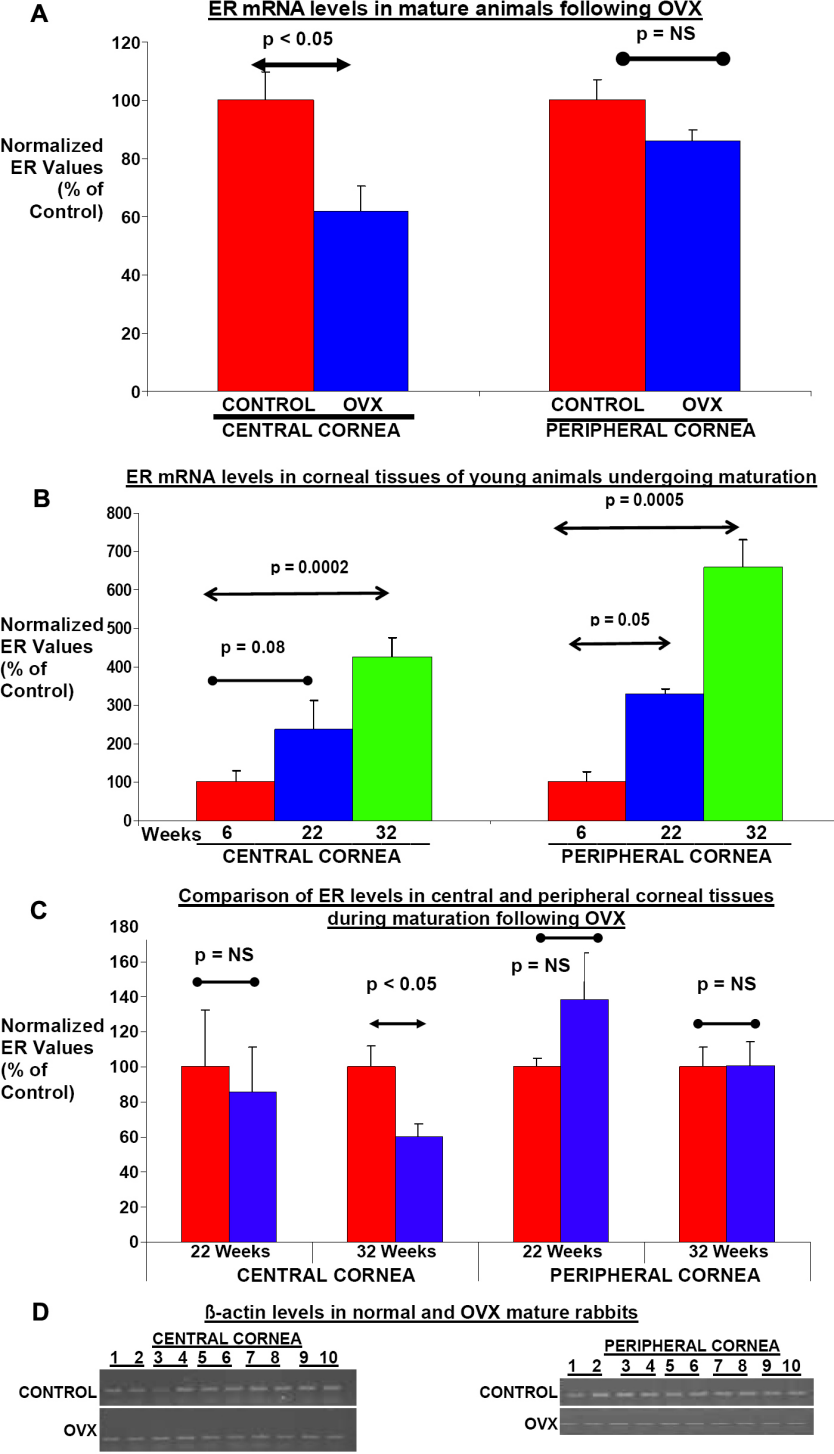
Expression of estrogen receptors in corneal tissues following ovariohysterectomy. ***A. Influence of OVX on ER mRNA levels in corneal tissues of skeletally mature animals:*** A cohort of skeletally mature 54 week old female rabbits were either left unoperated (controls) or were subject to ovariohysterectomy. After 8 weeks, cornea was collected from these animals and separated into central and peripheral corneal tissue using a 6 mm biopsy punch. mRNA levels of ER were determined by RT–PCR in these tissues. Values are plotted as a percentage of ER mRNA levels in normal control animals. Significant changes compared with normal controls are indicated (p<=0.05; p=not significant [NS]) **B. *ER levels in corneal tissues during maturation:*** Corneal tissues were collected from rabbits at 6, 22, and 32 weeks of age and separated into central and peripheral corneal tissues using a 6 mm biopsy punch. Levels of ER mRNA were determined by RT–PCR in these tissues. Values are plotted as a percentage of ER mRNA levels in 6 week old animals. Significant changes in ER levels compared with 6 week old animals are indicated (p<=0.05, p=not significant [NS]) ***C. Influence of pre-pubertal OVX on ER mRNA levels in corneal tissues:*** Eight week old rabbits underwent OVX surgery or were left unoperated to act as controls until 22 and 32 weeks of age. As described in the Methods section, corneal tissues were collected from these animals and mRNA levels for ER were determined by RT–PCR in these tissues. Values are plotted as a percentage of ER mRNA levels in normal control animals. Significant changes compared with normal controls are indicated (p<=0.05, p=not significant [NS]). ***D. mRNA levels for β-Actin remain unaffected by OVX:*** Central and peripheral corneal tissues were collected from both eyes of control (n=5) and OVX (n=5) skeletally mature 54-week-old female rabbits and were then examined for expression of the house keeping gene β-actin. mRNA levels of β-actin were determined by RT–PCR and then separated on a 2% agarose gel. Lanes 1–2, 3–4, 5–6, 7–8, and 9–10 represents data collected from the pair of eyes of 5 individual animals. The levels of the house keeping gene β-Actin remained unaffected by OVX.

## Methods

### Animals

One set of skeletally mature female NZW rabbits were obtained (Reiman's Furriers, St. Agatha, ON, Canada) at 52 weeks of age. Skeletal maturity in this source of rabbit occurs at 10–11 months of age based on radiographic assessments. The animals were housed locally in the Animal Care Facility in accordance with Canadian Council on Animal Care Guidelines and with the approval of the University of Calgary’s Animal Care Committee. The animals were allowed to acclimatize for 2 weeks before ovariohysterectomy (OVX). Of the total of 10 animals used in these experiments, 5 were subjected to surgical menopause induced by surgical removal of both uterus and ovary. Eight weeks following OVX, the control and experimental animals were sacrificed using an intravenous overdose of Euthanol (sodium pentobarbital; Abbott Laboratories, Montreal, Canada) and tissues were immediately collected and processed.

**Figure 2:**
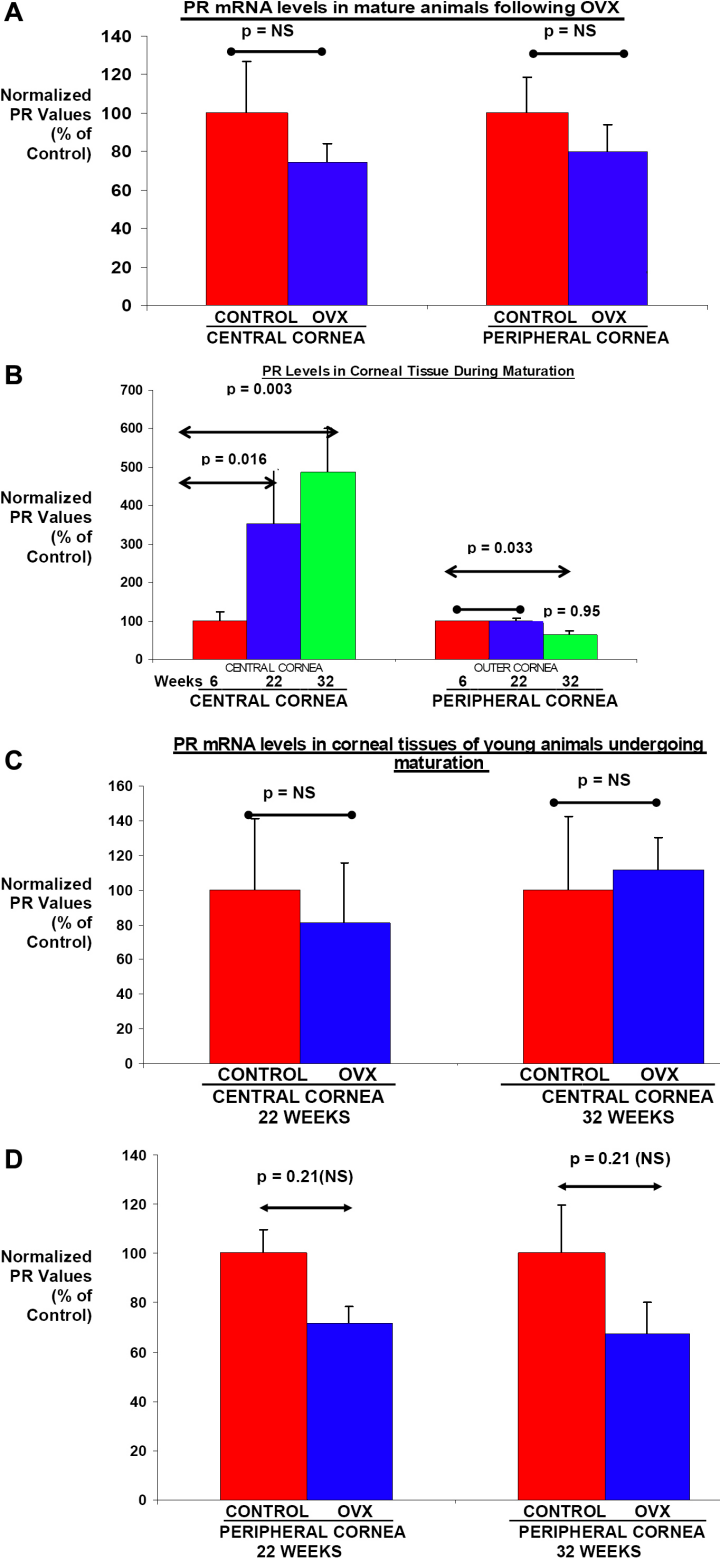
Expression of progesterone receptors in corneal tissues following ovariohysterectomy. **A: *PR mRNA levels in corneal tissues of skeletally mature animals following OVX:*** A cohort of skeletally mature 54-week-old female rabbits were either left unoperated (controls) or were subject to OVX. After 8 weeks, the cornea was collected from these animals and separated into central and peripheral corneal tissue using a 6 mm biopsy punch. mRNA levels of PR were determined by RT–PCR in these tissues. Values are plotted as a percentage of PR mRNA levels in normal control tissues. Significant changes compared with normal controls are indicated (p<=0.05, p=NS). **B: *Maturation associated changes in PR levels of corneal tissues:*** Corneal tissues were collected from rabbits at 6, 22, and 32 weeks of age and separated into central and peripheral corneal tissues using a 6 mm biopsy punch. Levels of PR mRNA were determined by RT–PCR in these tissues. Values are plotted as a percentage of PR mRNA levels in 6 week old animals. Significant changes in PR levels compared with the 6 weeks old animals are indicated (p<=0.05, p=NS). **C** and **D: *Influence of pre-pubertal OVX on PR mRNA levels in corneal tissues:*** Eight week old rabbits underwent OVX surgery or were left unoperated to act as controls until 22 and 32 weeks of age. As described in the Methods section, corneal tissues were collected from these 22 and 32 week old animals and mRNA levels for PR were determined by RT–PCR. Values are plotted as a percentage of PR mRNA levels in normal control animals. Significant changes compared with normal controls are indicated. (p<=0.05, p=not significant [NS]).

A second set of female NZW rabbits were obtained from the above mentioned source at 6 weeks of age. The animals were allowed to acclimatize for 2 weeks before ovariohysterectomy at 8 weeks of age. A cohort of 23 rabbits was used in this experiment. Of these, 5 rabbits were euthanized at 6 weeks of age to serve as pre-puberty controls. Of the 18 remaining rabbits, a cohort of 9 served as controls and 9 rabbits were subjected to surgical menopause by surgical removal of ovaries and uterus (OVX). The control and OVX animals were then euthanized at 22 (total n=8) and 32 (total n=10) weeks of age following the same protocol described above. The time course used in this study with sexually immature animals differed from that for the mature animals as this experiment focused on development related changes in gene expression following pre-pubertal ovariohysterectomy. As puberty in the rabbit occurs at 12–14 weeks of age, the intact animals would be sexually mature but not skeletally mature, and the surgically altered animals would not have experienced a normal puberty.

### Corneal tissue isolation

Following euthanization, corneal tissue was immediately collected from both eyes of the control and experimental animals by careful dissection to avoid contamination with limbus tissue. After removal, the corneas were washed in phosphate-buffered saline (PBS), and the central cornea was separated from the peripheral cornea using a 6 mm biopsy punch. Both the central and peripheral samples were frozen in liquid nitrogen, and then stored at −80 °C until processing. All samples in an experiment were subsequently processed at the same time to avoid potential variation.

### Molecular analysis

Total RNA was extracted from peripheral and central corneal tissues using the TRIspin method [20] and quantified using the SYBR Green reagent (Molecular Probes, Eugene, OR) method. Total RNA (1 μg) from each sample was initially reverse transcribed with random RT primers using a Qiagen Omniscript kit (Qiagen Inc.-Canada, Mississauga, Ontario, Canada) and then assessed by PCR using specific PCR-primers as detailed in Table 1. The protocol described previously was used throughout [21]. Amplicons were separated on a 2% agarose gel followed by staining with ethidium bromide. Quantity 1 1-D analysis (Bio-Rad Laboratories Ltd, Mississauga, Ontario, Canada) software was used to determine band density. By normalizing to the levels of the β-actin housekeeping gene (a gene not influenced by surgery), relative mRNA levels for the relevant molecules were determined. The analysis was then performed a second time with another aliquot of RNA and very similar results were obtained to those presented. Furthermore, recent studies have indicated that real time PCR analysis and results obtained as described above are comparable (unpublished data).

### Statistical analysis

Statistical analysis of the data was performed using ANOVA, STDEVA, and STEYX in Excel 5.0 software.

## Results

### Influence of surgical menopause on expression of steroid hormone receptors in central and peripheral corneal tissues

A cohort of 54-week-old female rabbits were either left unoperated (control) or were subjected to OVX to surgically remove the major hormone producing tissues. Corneal tissues were excised from these rabbits 8 weeks following OVX. The central and peripheral corneal tissues were separated and mRNA levels for estrogen receptor (ER) and progesterone receptor (PR) were examined using RT- PCR as described in the Methods section. The results presented in Figure 1A exhibit a mean ~39% decrease (p<0.05) in ER mRNA levels in the central corneal tissue following OVX. A modest but nonsignificant mean ~23% decrease (p>0.05; NS) was observed for PR mRNA levels in the same tissues following OVX (see Figure 2A). Interestingly, the peripheral corneal tissues exhibited no significant changes in ER and PR mRNA levels between control and OVX animals (Figure 1A and Figure 2A, respectively). These results suggest that cells in the central corneal tissues are highly responsive to fluctuations in estrogen levels and that low estrogen levels following OVX have a differential impact on ER mRNA levels in a location-specific manner. Furthermore, these results also suggest that as central and peripheral corneal tissues do not exhibit significant decreases in PR mRNA levels, these tissues are probably not as responsive to fluctuations in progesterone levels. Examination of mRNA levels for the housekeeping gene, β-actin, revealed no significant changes in its levels in central and peripheral cornea of control and OVX skeletally mature animals (Figure 1D).

In the second set of experiments, a group of animals were subjected to OVX before they reached puberty. Rabbits generally become sexually mature at the age of 12–14 weeks of age. Corneal tissues were collected from animals at 14 and 24 weeks after OVX when the animals were 22 and 32 weeks of age, respectively. In Figure 1B and Figure 2B, the ER and PR mRNA levels of the central and peripheral corneal tissues are displayed, respectively. ER mRNA levels were observed to increase with the age of the animals in both the central and peripheral corneal tissues. Thus, ER mRNA levels in the corneal tissue progressively increased from 6 to 32 weeks in the intact animals (Figure 1B). However, PR mRNA levels increased progressively from 6 to 32 weeks only in the central corneal tissues and not in the peripheral corneal tissues (Figure 2B). However, at 22 weeks, there were no significant differences in ER or PR mRNA levels in the central corneal tissues of age-matched control and OVX animals (see Figure 1C and Figure 2C). At 32 weeks of age however, the central corneal tissues did exhibit significant differences in ER mRNA levels between control and OVX animals (Figure 1C). There was a ~40% decrease in the ER mRNA levels in the central corneal tissues of the OVX animals. The PR mRNA levels in the central corneal tissues were not found to be significantly different from control values even at 32 weeks of age (Figure 2C).

The peripheral corneal tissues were also examined for ER and PR mRNA levels at 22 and 32 weeks of age. However, the ER or PR mRNA levels did not exhibit any significant differences when the peripheral tissues of control and OVX animals were compared at these time points (Figure 1C and 2D). These results once again suggest that peripheral corneal tissues are not as responsive to fluctuations in hormonal levels as are the central corneal tissues. Also, for this set of animals mRNA levels for the housekeeping gene, β-actin revealed no significant changes in levels in central and peripheral cornea following OVX (data not shown), similar to what was found for the mature animals (Figure 1D).

### Expression of collagens in the central and peripheral corneal tissues following OVX

The expression of some key forms of collagen such as collagen I, collagen III and collagen V was next examined in the central and peripheral corneal tissues with or without OVX, as collagens are the major structural component of the cornea [11,12]. Corneal tissues were harvested from control and OVX skeletally mature animals 8 weeks after the surgery and mRNA levels for collagens were analyzed using RT- PCR protocols.

**Figure 3:**
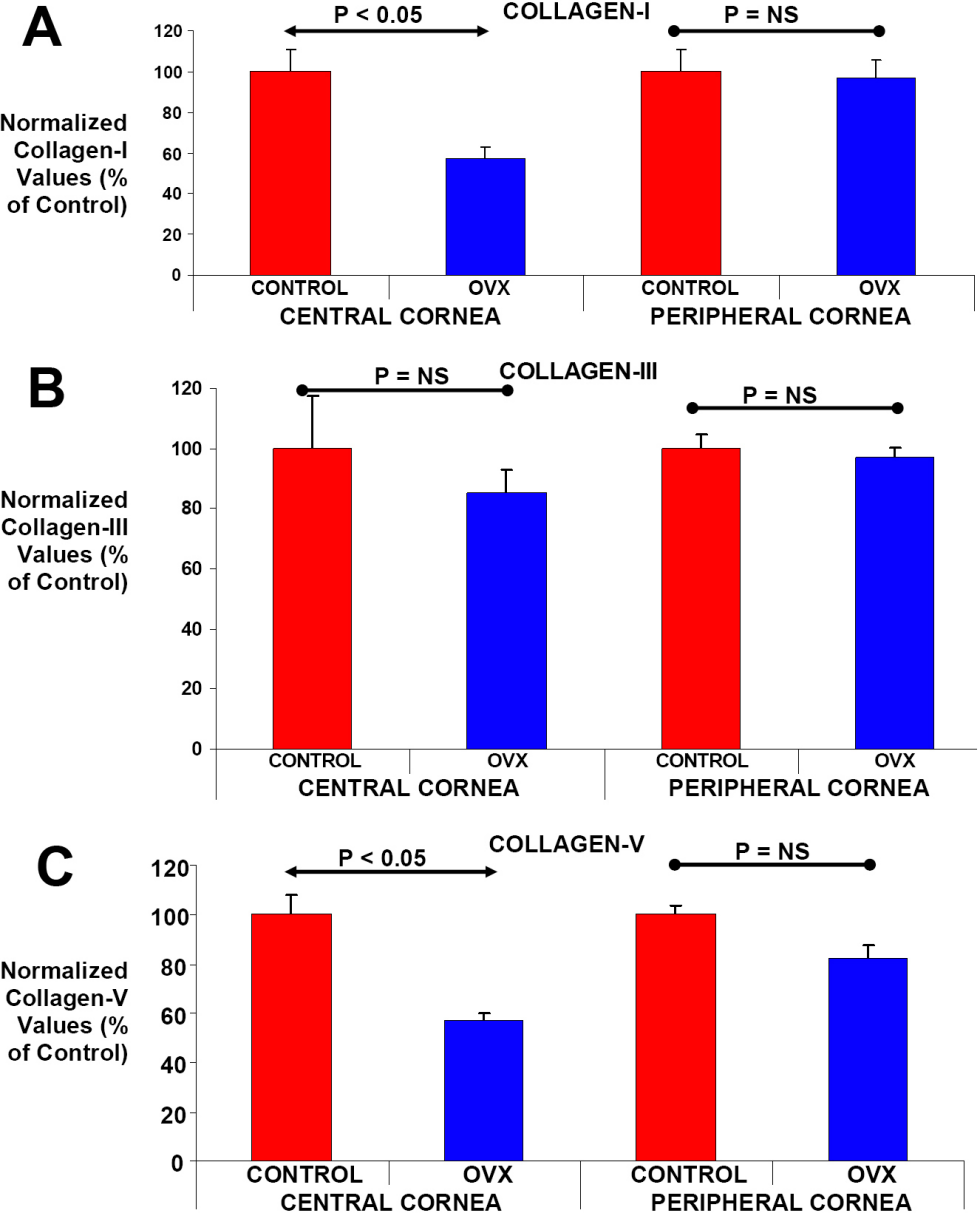
Comparison of collagen mRNA levels in peripheral and central corneal tissues following ovariohysterectomy. **A**, **B**, and **C;** Corneal tissues were collected from a cohort of 54-week-old control and ovariohysterectomized female rabbits. Corneal tissues were separated into peripheral and central parts by using a 6 mm biopsy punch. mRNA levels of collagen I, III, and V were determined by RT–PCR in these tissues. Values are plotted as a percentage of Collagen I, Collagen III, or Collagen V mRNA levels in normal control animals. Significant changes compared with normal controls are indicated (p<0.05, p=not significant [NS]).

As depicted in Figure 3A, collagen I mRNA levels decreased by ~43% in central corneal tissues of OVX animals when compared to control animals (p<0.05). Interestingly, collagen I mRNA levels were similar in the peripheral corneal tissues of control and OVX animals. Collagen V mRNA levels exhibited patterns of expression similar to those for collagen I in the central and peripheral corneal tissues obtained from control and OVX animals (Figure 3C). Collagen V mRNA levels also decreased by ~43% in the central corneal tissues of OVX animals when compared to controls (p<0.05). In contrast to collagen I and collagen V, collagen III mRNA levels did not exhibit significant decreases in either the central and peripheral corneal tissues of OVX animals when compared to control values (Figure 3B).

**Figure 4:**
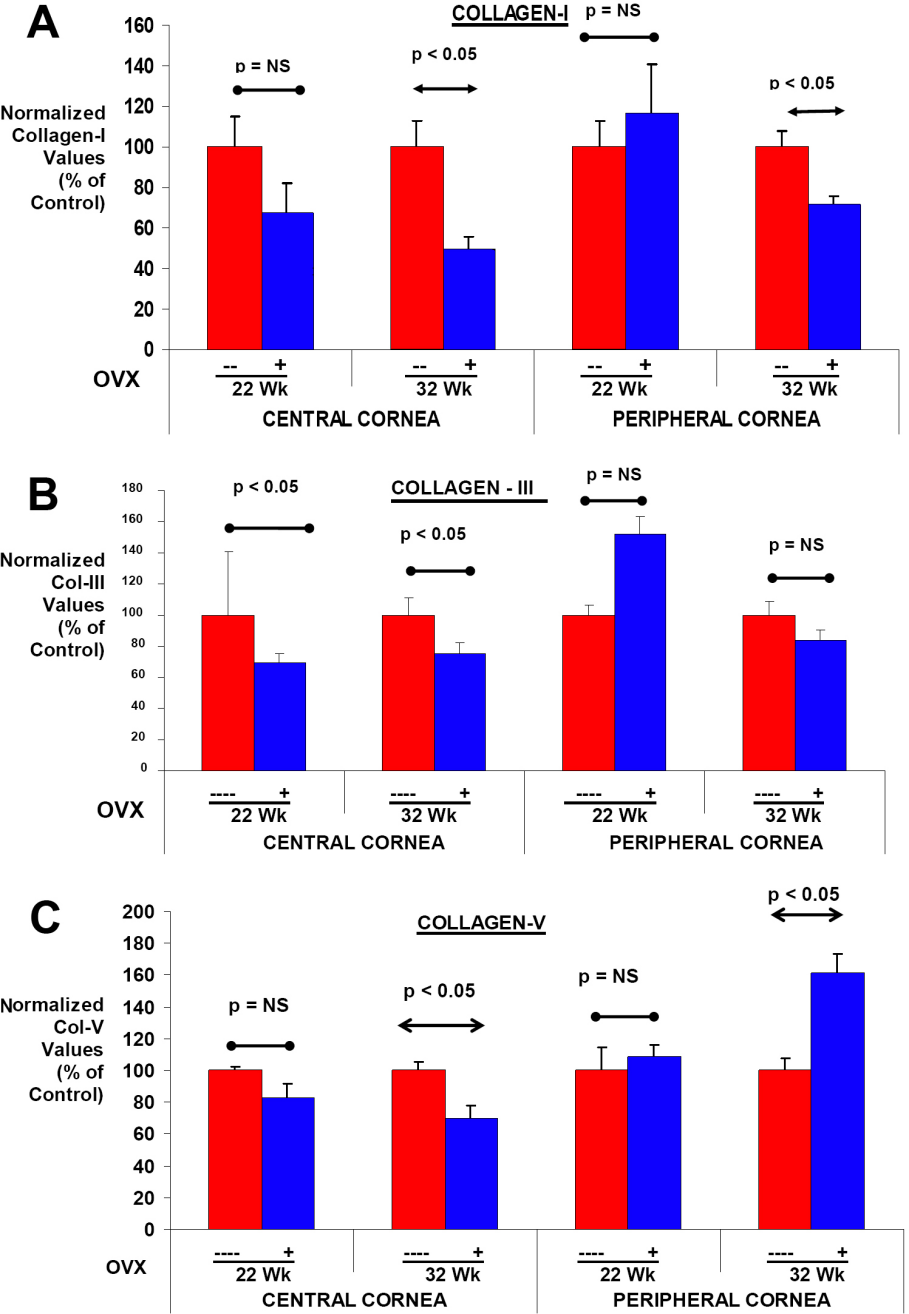
Influence of early OVX on the expression of collagen molecules in corneal tissues of immature rabbits. **A**, **B**, and **C:** A cohort of young female rabbits was either left unoperated (control) or subjected to OVX at 8 weeks of age. Corneal tissues were collected from these animals at 22 and 32 weeks of age, and central and peripheral corneal tissues were analyzed separately. Levels of collagen I, III, and V mRNA were determined by RT–PCR in these tissues. Values are plotted as a percentage of collagen I, III, or V mRNA levels in normal control animals. Significant changes compared with normal controls are indicated (p<=0.05, p=not significant [NS]).

Central and peripheral corneal tissues were also analyzed for collagen mRNA levels in the female rabbits which were subjected to OVX before they reached puberty. OVX rabbits at 22 wks of age (14 weeks post-OVX) did not exhibit significant differences in collagen I mRNA levels when compared to age-matched control values (Figure 4A). However, collagen I mRNA levels were decreased by ~50% in the central corneal tissue of the OVX rabbits at 32 weeks of age when compared to age matched control values (p<0.05 ; see Figure 4A). Interestingly, significant differences in collagen I mRNA levels were observed in the peripheral corneal tissues of OVX rabbits only at 32 weeks of age (p>0.05; Figure 4A).

Collagen III mRNA levels of the central corneal tissue of these rabbits did not vary significantly between control and OVX rabbits at 22 or 32 wks of age (Figure 4B). The patterns of expression of collagen V mRNA levels were again very similar to those for collagen I (compare Figure 4A and Figure 3C). OVX significantly affected collagen V mRNA levels only when the animals were older, at 32 weeks of age (Figure 4C). A mean 40% decrease was observed for collagen V mRNA levels in the OVX animals when compared to the age-matched control groups. Examination of the peripheral corneal tissues did not reveal any significant changes for collagen V mRNA levels at 22 wks of age (p>0.05; Figure 4C). However, in the same tissues the mRNA levels for collagen V were found to be significantly higher in OVX animals at 32 wks of age (p<0.05; Figure 4C).

### mRNA levels for small proteoglycans are differentially affected by low estrogen levels after surgical menopause

**Figure 5:**
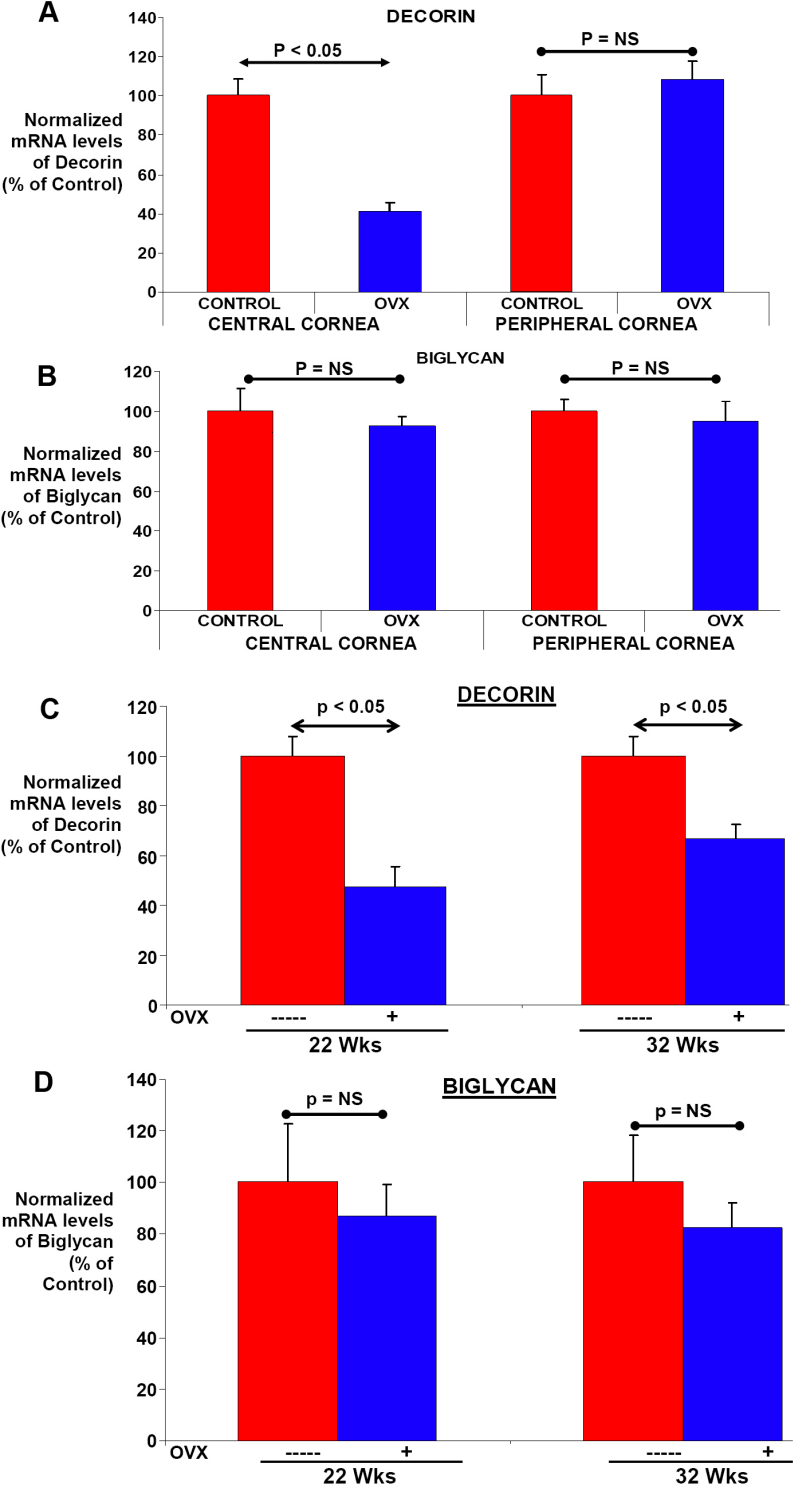
Influence of low estrogen levels on mRNA levels for biglycan and decorin. **A** and **B**: Corneal tissues were collected from a cohort of 54-week-old control and OVX female rabbits. Corneal tissues were separated into peripheral and central corneal tissues by using a 6 mm biopsy punch. The mRNA levels for proteoglycans such as decorin and biglycan were determined by RT–PCR in these tissues. **C** and **D**:. A cohort of young female rabbits was either left unoperated (control) or subject to OVX at 8 weeks of age. Corneal tissues were collected from these animals at 22 and 32 weeks of age and central and peripheral corneal tissues were analyzed separately. Levels of proteoglycan mRNA were determined by RT–PCR in these tissues. Values are plotted as a percentage of decorin or biglycan mRNA levels in normal control animals. Significant changes compared with normal controls are indicated (p<=0.05, p=not significant [NS]).

Subsequently, the influence of surgical menopause on mRNA levels for low molecular weight proteoglycans, specifically decorin and biglycan, were also evaluated. As shown in Figure 5A, decorin mRNA levels exhibited a significant mean ~59% decrease in the central corneal tissues of skeletally mature OVX animals. The peripheral corneal tissues did not exhibit any changes in decorin mRNA levels between the control and OVX animals. In contrast, mRNA levels for biglycan were not significantly altered (p>0.05) in either the central or the peripheral corneal tissues of control and OVX animals (Figure 5B).

mRNA levels for these proteoglycans were also examined in the corneal tissues of animals which were subjected to OVX before attaining sexual maturity. Interestingly, similar to the ER results (see Figure 1B), decorin mRNA levels exhibited age-dependent increases in normal animals as they matured from 6 weeks to 32 weeks of age (data not shown). At both 22 and 32 weeks of age, decorin mRNA levels were significantly lower (~34%) in the OVX animals when compared to controls (p<0.05, Figure 5C). The peripheral corneal tissues did not show any significant changes in the mRNA levels for decorin (data not shown). These results suggest that decorin mRNA levels in the central cornea are influenced by changes induced by OVX, either directly or indirectly, and occur more rapidly than those for the collagens in this experiment with younger animals.

In addition, biglycan mRNA levels were also examined in central corneal tissues of the same experimental animals. Biglycan mRNA levels in the central corneal tissues were similar between the control and OVX animals at both 22 and 32 wks of age (Figure 5D). The peripheral corneal tissues did not exhibit significant changes in mRNA levels for decorin and biglycan levels in control and OVX animals at different ages (data not shown). Thus, these two small proteoglycans, both known to interact with collagens [22], differ in their response to OVX in corneal tissues.

### mRNA levels for growth factors are influenced by surgical menopause

mRNA levels for several relevant growth factors were also analyzed from corneal tissues. These tissues were obtained from the skeletally mature rabbits which had been subjected to OVX when mature. The results presented in Table 2 show that basic fibroblast growth factor (FGF), connective tissue growth factor (CTGF), insulin –like growth factor 1 (IGF-1), nerve growth factor (NGF), and transforming growth factor beta 1 (TGF-β1), all exhibited mean decreases of ~40%–50% in mRNA levels 8 weeks following OVX in the central cornea. Of the six growth factors that were assessed, only vascular endothelial growth factor (VEGF) did not exhibit significant differences in central corneal tissues of control and OVX animals, but this may not be surprising since the central cornea is somewhat avascular [23,24]. Examination of peripheral corneal tissues did not exhibit significant differences in mRNA levels for these growth factors in both the control and OVX animals (data not shown).

Furthermore, mRNA levels for these growth factors were also examined in central corneal tissues of animals subjected to OVX in the pre-puberty state. As shown in Table 2, mRNA levels for these growth factors were approximately the same (p>0.05; NS) in central corneal tissues of control and OVX animals at 22 weeks of age. However, there were significant decreases in the expression of most of these molecules in central corneal tissues of OVX animals, detected when the animals were 32 weeks of age (Table 2). Interestingly, mRNA levels for VEGF were again not significantly impacted by pre-puberty OVX at either 22 or 32 weeks of age. Furthermore, both adult and young animals show a similar response pattern for VEGF mRNA.

### Influence of low hormonal levels on mRNA values for CATHK, TSP-1, and OPN

Subsequently, central corneal tissues were also examined for osteopontin (OPN), thrombospodin 1(TSP-1), and cathepsin K (CATHK) in mature animals with or without OVX. Osteopontin and CATHK play important roles in the synthesis and degradation of collagen, the principal constituent of cornea, and TSP-1 is essential for maintaining its avascularity. In central corneal tissues, mRNA levels for CATHK and OPN significantly declined (~78%–45%) eight weeks after surgery (Table 3). However no significant changes were observed for TSP-1 under the same conditions. Thus, both TSP-1 and VEGF exhibit a similar lack of response to OVX.

Furthermore, the expression of these molecules was also examined in central corneal tissues from pre-puberty OVX animals. When the animals were 22 weeks old, there were no significant alterations in mRNA levels for either CATHK, OPN, or TSP-1 (Table 3) induced by OVX. Examination of central corneal tissues from the 32 week old animals also did not identify any changes in mRNA levels for OPN and TSP-1, but mRNA levels for CATHK were again decreased by ~50% (Table 3).

Peripheral corneal tissues did not exhibit significant differences for these three molecules in controls and either pre-pubertal OVX animals or skeletally mature OVX animals (data not shown).

## Discussion

This in vivo study focused on elucidating possible changes in mRNA level patterns in corneal tissues following menopause, when the of sex-hormones are low. The primary observations presented here suggest that mRNA levels for several different molecules such as ER, decorin, collagen 1, collagen V, and several growth factors decreased in the “post-menopausal” period, but primarily only in the central cornea. Interestingly, the observed changes in these mRNA level patterns in the central corneal tissues were found to be influenced by the age of the animals and timing of the surgery. The decline in the levels of gene expression in corneal tissues proved to be rapid in the mature adult animals, occurring by 8 weeks post-OVX surgery. In contrast, OVX surgery in the pre-pubertal stage had a significantly delayed impact as molecular alterations were observed primarily at 24 weeks post-surgery when the animals were 32 weeks of age. Interestingly, the absence of alterations in mRNA levels in corneal tissues of adolescent animals early after being subjected to pre-pubertal OVX surgery suggests that growth and maturation parameters may override the influence of low hormonal conditions and the impact is only observed when growth related stimuli decline.

The findings presented in this study also demonstrate that only a subset of molecules is responsive to surgically induced “menopausal” conditions. Interestingly, nearly the same set of molecules was impacted in corneal tissues of both young and adult animals following surgery. For example, biglycan was not impacted by OVX in either adult or pre-pubertal menopausal animals, whereas decorin, a member of the same proteoglycan family, was impacted by the low hormonal conditions in both young and adult animals. Furthermore, the extent of the decreases in mRNA levels for the affected molecules was in the range of ~40%–60% for most of OVX sensitive molecules, irrespective of the age of the animals. Thus, in both experimental circumstances, a unique subset of molecules was affected, and to nearly the same extent.

For these studies, the corneal tissues were divided into peripheral and central cornea. Central cornea is the area of the tissue which covers the pupil of the eye. The cornea is a transparent, fairly avascular structure which modulates the optical power of the eye in combination with the lens. Interestingly, central corneal tissues exhibited more extensive alterations in mRNA profiles following surgery and thus proved to be a highly responsive area when compared to peripheral corneal tissue. Several differences have been elucidated between central and peripheral cornea. Topographically, peripheral cornea is flatter than central cornea. This marked change in the topography has been attributed to the circumferential arrangement of the collagen fibrils in the peripheral corneal region. Moreover, peripheral corneal thickness is asymmetric and is reported to vary with age [25] and with menstrual cycle hormonal changes in women [26]. In addition, cells of the peripheral cornea are reported to be highly proliferative when compared to those of central cornea [27]. Previous studies have demonstrated that the pre-pupillary (central) portion of the cornea differs with respect to mRNA expression levels from the peripheral area of the cornea for several connective-tissue molecules in a rabbit model [28] and the present studies confirm and extend these findings.

Previous studies have demonstrated the presence of estrogen and progesterone receptors in the cornea of rats, rabbits, and human [10]. In the present study, examination of the expression of estrogen receptors in corneal tissues revealed a mean ~40% decrease in ER mRNA levels in central corneal tissues of OVX animals as compared to controls. Moreover, peripheral corneal tissues did not exhibit significant differences in ER mRNA levels under the same conditions. PR mRNA levels did not decrease significantly in the central or the peripheral cornea of OVX animals suggesting that corneal tissues are perhaps more influenced by estrogen than progesterone. Similar trends were also detected for mRNA profiles for other molecules examined in this study such as collagen 1, collagen V, and several growth factors such as bFGF, CTGF, NGF, IGF-1, and TGF-β. These results suggest that cells in the central cornea are more responsive to hormonal fluctuations than those in peripheral corneal tissues, particularly at the mRNA level.

Collagen fibrils form the principal structural component of the cornea and are closely packed in the central cornea [29]. The stroma of normal human cornea is rich in type I and type V collagens and mRNA levels for both molecules exhibited a ~40%–50% decrease in their expression in central cornea, and a ~30% decrease in collagen I mRNA levels in peripheral cornea at 32 weeks of age. Interestingly, collagen V levels were uniquely elevated in the peripheral cornea tissues when animals were also 32 weeks of age. This anomaly occurred only at this time point and was not observed in skeletally mature animals. The basis for this change should be further investigated. In contrast, collagen III levels did not exhibit significant differences in either the central or peripheral corneal tissues following OVX. Collagen III is generally present at low levels in normal tissues, but increases during wound healing, inflammation and several pathological conditions [30].

**Table 2:** mRNA levels for different growth factors in central corneal tissues following ovariohysterectomy.

**Mature animals**			
**Growth factors**	**Control**	**OVX**	**p-Value**
bFGF	100	52.4	0.0007
CTGF	100	62.2	0.00002
IGF-1	100	54.5	0.02
NGF	100	54.4	0.00001
TGF-β	100	62.0	0.002
VEGF	100	102.0	NS
**Immature animals**			
Age of rabbits	22 weeks		
**Growth factors**	**Control**	**OVX**	**p-Value**
bFGF	100	82.9	NS
CTGF	100	110.4	NS
IGF-1	100	133.7	NS
NGF	100	87.7	NS
TGF-β	100	70.3	NS
VEGF	100	76.4	NS

Age of rabbits	32 weeks		
**Growth Factors**	**Control**	**OVX**	**p-Value**
bFGF	100	70.6	<0.05
CTGF	100	50.7	<0.05
IGF-1	100	78.2	<0.05
NGF	100	69.4	<0.05
TGF-β	100	61.8	<0.05
VEGF	100	117.7	NS

Corneal tissues were collected from a cohort of skeletally mature and immature control and ovariohysterectomized (OVX) female rabbits. Tissue was collected at 2 months post-OXV from mature animals, and at 22 and 32 weeks of age (14 and 24 weeks post-OVX, respectively) for the immature animals. Corneal tissues were separated into central and peripheral areas using a 6 mm biopsy punch and mRNA levels for a subset of growth factors were determined by RT–PCR as described in the Materials and Methods section. Control values were set at 100%. Mean experimental values are only displayed for the central corneal tissues as the peripheral tissues did not exhibit any significant changes following OVX. Significant changes compared with normal controls are indicated (p<0.05 (significant), p=not significant [NS]).

It is well known that corneal transparency requires uniform spacing of the collagen fibrils and proteoglycans are reported to play an important role in this process [31,32]. These macromolecules are a major component of the corneal stroma where they function to maintain hydration and the structural organization of the tissue [33]. In the present study, mRNA levels for two collagen-binding proteoglycans, decorin and biglycan, have been evaluated. Decorin mRNA levels were found to be significantly depressed in the central cornea of both adult and pre-pubertal OVX animals when compared to controls (Figure 5), but not in peripheral corneal tissues.

Interestingly, biglycan mRNA levels were not affected by OVX in either experiment, in the central or peripheral cornea. Interestingly, decorin is predominantly found in the corneal stroma [34], while biglycan has been identified in the corneal epithelium [35]. Although the detectable mRNA levels for biglycan in the normal rabbit cornea are very low, its levels are markedly increased in some pathological conditions and in corneal scars [36,37]. TSP1 is another multifunctional, matricellular glycoprotein which showed responses similar to biglycan in corneal tissues following OVX in young and mature animals. TSP-1 is an anti-angiogenic factor thought to be involved in maintaining corneal avascularity [38,39] and it is reported to be expressed at higher levels in the corneal epithelium [38]. The lack of significant alterations in the expression levels of TSP-1, biglycan, and collagen III in response to OVX suggests that a unique subset of genes are not as sensitive to changes in hormone levels, at least in rabbit corneal tissues. However, this conclusion does not rule out the possibility that the impact of low hormonal conditions on variations in mRNA levels might exhibit tissue or cell specific behavior. Interestingly, these four non-responsive genes (collagen III, biglycan, TSP-1, and VEGF) are all reported to be associated with wound healing processes [30].

In general, growth factors also play a crucial role in tissue maintenance, as well as wound healing and repair of corneal tissues [40]. It has been reported that normal corneal functioning requires the production of growth factors by the corneal epithelium, stroma, and endothelium, as well as the adjacent tear layer [40]. In the present study, the mRNA levels for several relevant growth factors in control and OVX animals was examined and almost all of those assessed exhibited significant decreases following surgical menopause.

Furthermore, examination of molecules such as Cathepsin K and OPN also revealed a decrease in mRNA levels in corneal tissues following OVX in both pre-pubertal and adult animals. Cathepsin K has been shown to degrade collagens [41]. Osteopontin is a glycosylated protein primarily located in the extracellular matrix and body fluids of various tissues and it can modulate collagen fibrillogenesis. Thus, the finding that OVX leads to depressed mRNA levels for these genes may indicate that maintenance and repair processes become compromised after menopause, and that adequate hormone levels are required for sustaining proper corneal function and preventing age-related declines.

Interestingly, VEGF mRNA levels did not exhibit significant differences between central corneal tissues of control and OVX animals. The avascularity of the cornea is essential for maintaining its transparency and, consequently, clearer vision. The molecular mechanisms responsible for maintaining avascularity of the cornea are complex and poorly elucidated. Paradoxically, VEGF, which is a potent stimulator of angiogenesis, is present in avascular corneal tissue [23] and recent studies have implicated the necessity of soluble VEGF receptor-1 for corneal avascularity. Another study has demonstrated that VEGF receptor 3 is constitutively expressed in corneal tissues and perhaps results in suppression of angiogenesis, thereby contributing to corneal avascularity tissue [23].

**Table 3:** Changes in mRNA levels for CathK, OPN and TSP-1 in the central cornea of rabbits following ovariohysterectomy.

**Mature animals**			
**Protein**	**Control**	**OVX**	**p-Value**
Cathepsin K	100	61.2	0.0006
Osteopontin	100	55.3	0.0008
Thrombospondin 1	100	82.6	NS
**Immature animals**			
Age of rabbits	22 weeks		
**Protein**	**Control**	**OVX**	**p-Value**
Cathepsin K	100	72.6	NS
Osteopontin	100	78.4	NS
Thrombospondin 1	100	136.8	NS

Age of rabbits	32 weeks		
**Protein**	**Control**	**OVX**	**p-Value**
Cathepsin K	100	42.8	<0.05
Osteopontin	100	73.9	NS
Thrombospondin 1	100	95.5	NS

Corneal tissues were collected from a cohort of skeletally mature and immature control and ovariohysterectomized (OVX) female rabbits as described in Table 2. Corneal tissues were separated into central and peripheral areas using a 6 mm biopsy punch and mRNA levels for the indicated molecules determined by RT–PCR. Only values for the central corneal tissues are presented and are given as mean percentage of cathepsin K, osteopontin or thrombospondin 1 mRNA levels in normal control tissues. Significant changes compared with normal controls are indicated (p<0.05 (significant), p=not significant [NS]).

In conclusion, this in vivo study clearly demonstrated declining mRNA levels for a subset of genes following “menopause” in adult corneal tissues. Furthermore, deviating onset of puberty also appears to have long-term impact on the expression of a similar subset of molecules. Interestingly, decreases in mRNA levels in the central cornea were observed only when animals subjected to pre-puberty OVX reached 32 weeks of age, with minimal impact on mRNA levels when the animals were only 22 weeks of age. As these animals are continuing to mature at 22 weeks of age, but such growth influences are declining by 32 weeks of age, growth stimuli may override the loss of puberty-associated hormonal influences when the animals are young. Relevant to this discussion are previous reports which suggest that pre-pubertal neutering leads to a delay in the closure of growth plates, as sex hormone increases during puberty are essential for this process. Therefore, animals subjected to pre-pubertal OVX tend to grow more than their normal counterparts [42,43]. However, we did not detect significant influences on rabbit weights after OVX (unpublished observations). Furthermore, a study on the influence of pre-pubertal OVX in a canine model has demonstrated that the bone mineral density and bone mineral content were only impacted once the animals were 6 months of age [44]. In contrast, the lowering of hormone levels following menopause in a skeletally mature animal appears to exert a more rapid onset on mRNA levels for a specific subset of genes. In the present study, significant decreases in mRNA levels for this subset were observed by 8 weeks after OVX in skeletally mature animals subjected to OVX, and to achieve the same lower levels of expression in animals subjected to OVX before puberty took 24 weeks. These results suggest that impact of estrogen removal is more rapid in skeletally mature adult female animals than in younger female rabbits.

The normal process of aging in women is accompanied by depression in hormonal levels due to the onset of menopause. Corneal changes in mRNA levels following OVX presented here provide new insights into the regulation of gene expression in the cornea, insights which may be relevant to events following menopause. Future directions may involve replenishing OVX animals with either estrogen or progesterone, or both, to assess whether mRNA levels for hormone influenced genes can be restored. In addition, it will be important in the future to assess if only a subset of cells in central cornea are being affected by OVX, and finally studies to confirm the observed alterations in mRNA levels are reflected by changes in protein expression and tissue levels need to be performed.
